# Local heating increases skin gas acetone content independent of systemic metabolism

**DOI:** 10.1038/s41598-026-49166-y

**Published:** 2026-04-18

**Authors:** Keito Kiriyama, Naoya Okumura, Kentaro Taniguchi, Norio Hotta, Makoto Sawano

**Affiliations:** 1 College of Life and Health Science, Chubu University, Kasugai, Aichi Japan; 2 Faculty of Human Relations, Tokai Gakuin University, Kakamigahara, Gifu Japan; 3 National Cerebral and Cardiovascular Research Center, Suita, Osaka Japan; 4 Department of Medical Laboratory Science, Faculty of Health Sciences, Kochi Gakuen University, Kochi, Japan; 5 Center for Advanced Emergency Medicine and Critical Care, Saitama Medical Center, Kawagoe, Japan

**Keywords:** Skin gas, Acetone, Local heating, Noninvasive monitoring, Volatile organic compounds, Biochemistry, Biomarkers, Health care, Medical research, Physiology

## Abstract

Skin-emitted volatile organic compounds have attracted attention as noninvasive biomarkers of physiological and metabolic states. However, the factors regulating the release dynamics of skin gases remain poorly understood. Here, we investigated the effects of local skin heating on the acetone concentration in skin gas using breath acetone as a reference for systemic metabolism. Skin gas and breath samples were collected from healthy adult males during a 30-min local heating protocol applied to the hands and forearms. Acetone levels were measured using sensor gas chromatography, and physiological variables, including skin temperature, skin blood flow, and blood β-hydroxybutyrate concentration, were assessed. Local heating significantly increased the skin gas acetone levels at the heated site. Notably, the skin gas acetone levels significantly exceeded the breath acetone levels during short-term heating. However, blood β-hydroxybutyrate concentration did not change significantly. Increased skin acetone levels at the heated site were significantly associated with increased skin temperatures and blood flow. These findings indicate that skin gas acetone responds dynamically to local physical and circulatory changes independent of systemic metabolic alterations. Our study provides insights into the temperature-dependent and site-specific behavior of skin gas acetone and supports its potential for noninvasive biomonitoring.

## Introduction

Volatile organic compounds (VOCs) are continuously released from the human body through breath, sweat, urine, and the skin^1,2^. These VOCs contain information reflecting metabolic processes and pathological conditions and have therefore attracted considerable attention as noninvasive tools for physiological and pathological assessment^3^. In particular, the applicability of breath VOCs for the early diagnosis and monitoring of diseases such as diabetes, cancer, and infectious diseases^4–7^ has been extensively investigated, with a major focus on biogas analysis.

In recent years, attention has also turned to a wide variety of gaseous compounds, collectively referred to as skin gases, which are continuously emitted from the human skin. Skin gas contains trace levels of VOCs and inorganic gases, typically in the parts per billion (ppb) to parts per trillion (ppt) range. Similar to breath, skin gas contains physiological information that can be collected noninvasively. Skin gas measurements do not require respiratory control and allow for continuous sampling under resting conditions. Owing to these characteristics, skin gas has been recognized as a useful indicator for biomedical monitoring^8^.

Skin gas emission pathways are broadly classified into three categories: surface reaction-derived, skin gland-derived, and blood-derived^9–12^. Surface- and gland-derived components originate from local biochemical processes on or within the skin. In contrast, blood-derived components originate from volatile substances in the bloodstream that diffuse through skin tissues and are emitted into the ambient air. Acetone, a representative ketone body, is detected in skin gas via the blood-derived pathway^13,14^. Although this pathway is widely assumed to reflect passive diffusion driven by concentration gradients between blood and the external environment, its quantitative dynamics and responsiveness to physical factors, such as local heating, remain insufficiently characterized. Additionally, the skin is increasingly being recognized as a neuro–immuno–endocrine organ that actively responds to environmental stimuli and contributes to systemic homeostasis^15^. This integrated regulatory system may influence the production, transport, and release of volatile compounds from the skin, suggesting that skin gas emissions are not governed solely by passive diffusion processes.

Acetone is primarily produced in the liver from acetyl-CoA generated during fatty acid β-oxidation and is formed via the non-enzymatic decarboxylation of acetoacetate^16^. Acetone levels in biogas increase under conditions of enhanced lipid metabolism, such as fasting, diabetes, and post-exercise states, and have therefore been used as indicators of lipid metabolic dynamics^17,18^. Skin gas acetone concentrations have been reported to show significant correlations with blood β-hydroxybutyrate levels^19^, suggesting the potential of skin gas analysis for metabolic monitoring. Moreover, acetone is a relatively abundant and stable component of skin gas, and its correspondence with breath acetone levels has been reported previously^2,20^. Therefore, acetone is considered a representative model compound for skin gas research.

However, most previous studies on skin gases have primarily focused on the identification and compositional analysis of the emitted compounds, whereas quantitative and systematic investigations of the factors governing skin gas emissions remain limited. As skin gas concentrations are generally lower than those in the breath, they are susceptible to environmental and measurement conditions. However, the effects of physical and circulatory factors, such as temperature and skin blood flow, on skin gas emission dynamics remain poorly understood.

Local heating increases skin temperature and induces vasodilation, thereby enhancing skin blood flow and altering metabolic activity and substance permeability within skin tissues^21,22^. In addition, an elevation in skin surface temperature may facilitate the release of low-molecular-weight volatile compounds, suggesting that local heating may influence skin gas emission dynamics. Therefore, examining the changes in skin gas release induced by local heating will be useful for understanding the influence of physical and circulatory factors on skin gas behavior.

We hypothesized that local heating would primarily enhance acetone emissions from the skin via local thermal and circulatory mechanisms rather than through systemic changes in lipid metabolism. Accordingly, we aimed to determine whether local heating increases skin gas acetone levels and clarify the underlying mechanisms by comparing heated and non-heated sites using breath acetone as a systemic reference. In addition to skin gas collected from heated and non-heated sites, breath acetone levels were measured as indicators of systemic metabolic status. By comparing these measurements over time, we sought to clarify the temperature dependence and local characteristics of acetone emissions from the human skin.

Elucidating the local determinants of skin gas emissions may advance our physiological understanding of skin-derived volatile compounds and contribute to the development of more reliable and standardized approaches for non-invasive metabolic monitoring in both research and clinical settings. These insights are essential for appropriately interpreting skin gas measurements under varying environmental and thermal conditions.

## Results

### Time-dependent changes in acetone levels across sampling sites

Twenty-one healthy adult males were subjected to localized heating (41 °C, 30 min) of the right hand and distal forearm after baseline sampling under seated conditions. Skin gas was collected from heated and non-heated sites at the baseline and at 10, 20, and 30 min with concurrent breath sampling.

**Fig. 1:**
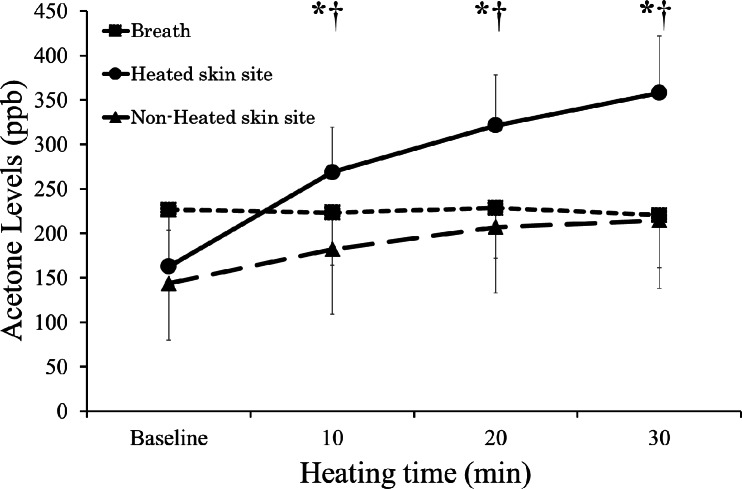
Time-dependent changes in acetone levels during local heating at the heated site, non-heated site, and in exhaled breath. The acetone levels at each measurement site are shown at baseline (before heating) and during local heating (10, 20, and 30 min). Data are presented as mean values with error bars indicating standard deviation (SD) (*n* = 21). A two-way repeated-measures analysis of variance revealed significant main effects of measurement site (F (2, 40) = 17.586, *p* < 0.01, partial η² = 0.468) and time (F (3, 60) = 280.181, *p* < 0.01, partial η² = 0.933), as well as a significant interaction between site and time (F (6, 120) = 106.911, *p* < 0.01, partial η² = 0.842). An asterisk (*) indicates a significant difference between the heated and non-heated sites at the same time point (*p* < 0.01), and a dagger (†) indicates a significant difference between the heated site and exhaled breath at the same time point (*p* < 0.01).

The time-dependent changes in acetone levels measured at heated and non-heated skin sites and in the breath are summarized in Fig. 1. Two-way repeated-measures analysis of variance (ANOVA) with Greenhouse–Geisser correction was performed because of a violation of sphericity.

The two-way repeated-measures ANOVA revealed significant main effects of sampling site (F (2,40) = 17.586, *p* < 0.01, partial η² = 0.468) and time (F (3,60) = 280.181, *p* < 0.01, partial η² = 0.933), as well as a significant interaction between sampling site and time (F (6,120) = 106.911, *p* < 0.01, partial η² = 0.842).

At the heated skin site, the acetone concentration increased significantly over time (*p* < 0.01). At the nonheated skin site, a gradual and significant increase in acetone levels was observed (*p* < 0.01). In contrast, no significant time-dependent changes were observed in breath acetone levels.

At the baseline, breath acetone levels were highest among the three sampling sites. However, acetone levels at the heated skin site exceeded those in the breath 10 min after the onset of heating, and this relationship persisted until 30 min.

### Changes in acetone levels at the heated and non-heated skin sites

To examine whether the observed site-specific differences were influenced by systemic acetone fluctuations, a linear mixed-effects model was constructed, using breath acetone levels as time-dependent covariates. In the primary model without covariate adjustment, the site × time interaction was statistically significant (F (3,160) = 7.02, *p* < 0.001). After adjusting for breath acetone, the site × time interaction remained statistically significant (F (3, 159) = 7.12, *p* < 0.001). Breath acetone level was not a significant independent covariate (*p* = 0.074).

### Associations between skin gas acetone levels and physiological variables

**Fig. 2:**
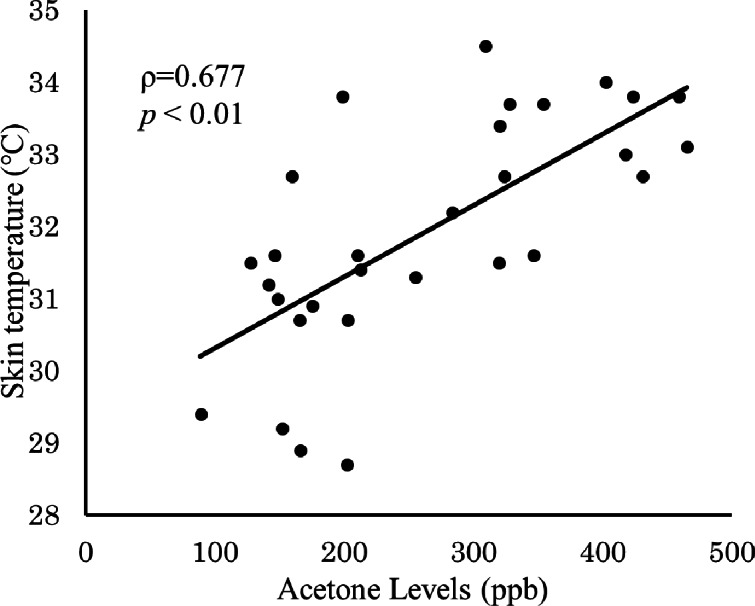
Correlation between skin gas acetone levels and skin temperature at the heated site. Measurements obtained at baseline (before heating) and 30 min after local heating were analyzed (*n* = 15). The correlation coefficients and the corresponding *p*-value are shown in the figure.

**Fig. 3:**
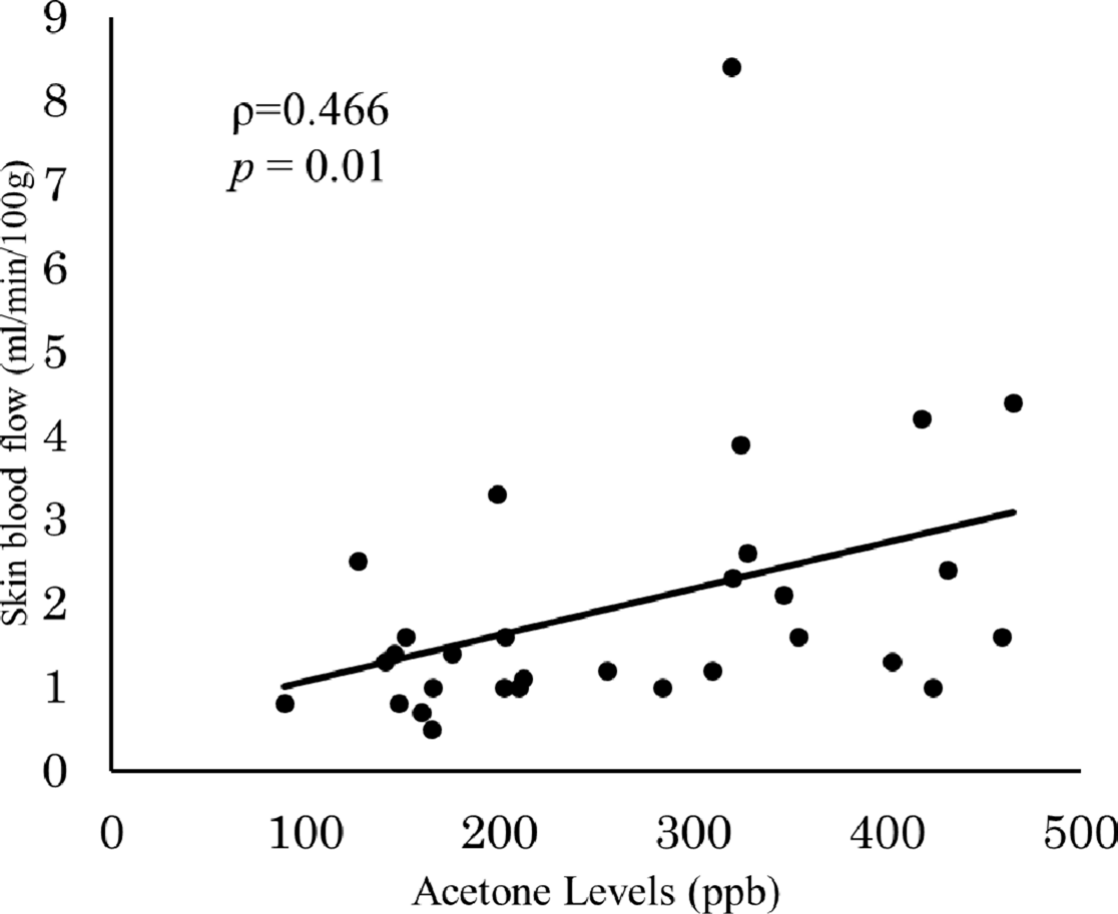
Correlation between skin gas acetone levels and skin blood flow at the heated site. Measurements obtained at baseline (before heating) and 30 min after local heating were analyzed (*n* = 15). The correlation coefficients and corresponding *p*-value are shown in the figure.

In a subset of 15 participants, physiological parameters, including skin temperature, skin blood flow, and blood β-hydroxybutyrate concentration, were measured before baseline sampling and after the 30-min heating protocol. The mean values of skin temperature, skin blood flow, and blood β-hydroxybutyrate concentration before and after local heating are shown in Table 1. At the heated skin site, acetone levels showed a significant positive correlation with skin temperature (ρ = 0.677, *p* < 0.01; Fig. 2) and skin blood flow (ρ = 0.466, *p* = 0.01; Fig. 3). No significant correlation was observed between acetone levels and blood β-hydroxybutyrate concentration.

**Table 1:** Skin temperature, skin blood flow, and blood β-hydroxybutyrate concentrations before and after local heating.

	Heated skin site (baseline)	Heated skin site (post-heating)	Non-Heated skin site (baseline)	Non-Heated skin site (post-heating)
	Mean ± SD	Mean ± SD	Mean ± SD	Mean ± SD
Skin temperature (℃)	30.7 ± 1.1	33.2 ± 0.9*†	30.6 ± 1.3	31.8 ± 1.1*
Skin blood flow (mL/min/100 g)	1.2 ± 0.5	2.8 ± 1.9*	1.2 ± 0.6	1.8 ± 0.7*
Blood β-hydroxybutyrate (mmol/L)	0.1 ± 0.0	0.1 ± 0.0	0.1 ± 0.0	0.1 ± 0.0

Values are presented as the mean ± standard deviation (SD) (*n* = 15). An asterisk (*) indicates a significant difference between before and after heating at the same site (*p* < 0.05), and a dagger (†) indicates a significant difference between the heated and non-heated sites at the same time point (*p* < 0.05). All comparisons were performed using Wilcoxon signed-rank tests.

Multiple linear regression analysis demonstrated that the overall model was significant (F (3,26) = 10.63, *p* < 0.01, R² = 0.551). Among the independent variables, skin temperature was a significant predictor of acetone levels (β = 0.609, *p* < 0.01), whereas skin blood flow showed a tendency toward significance (β = 0.269, *p* = 0.061). The blood β-hydroxybutyrate concentration did not contribute significantly to the model.

**Fig. 4:**
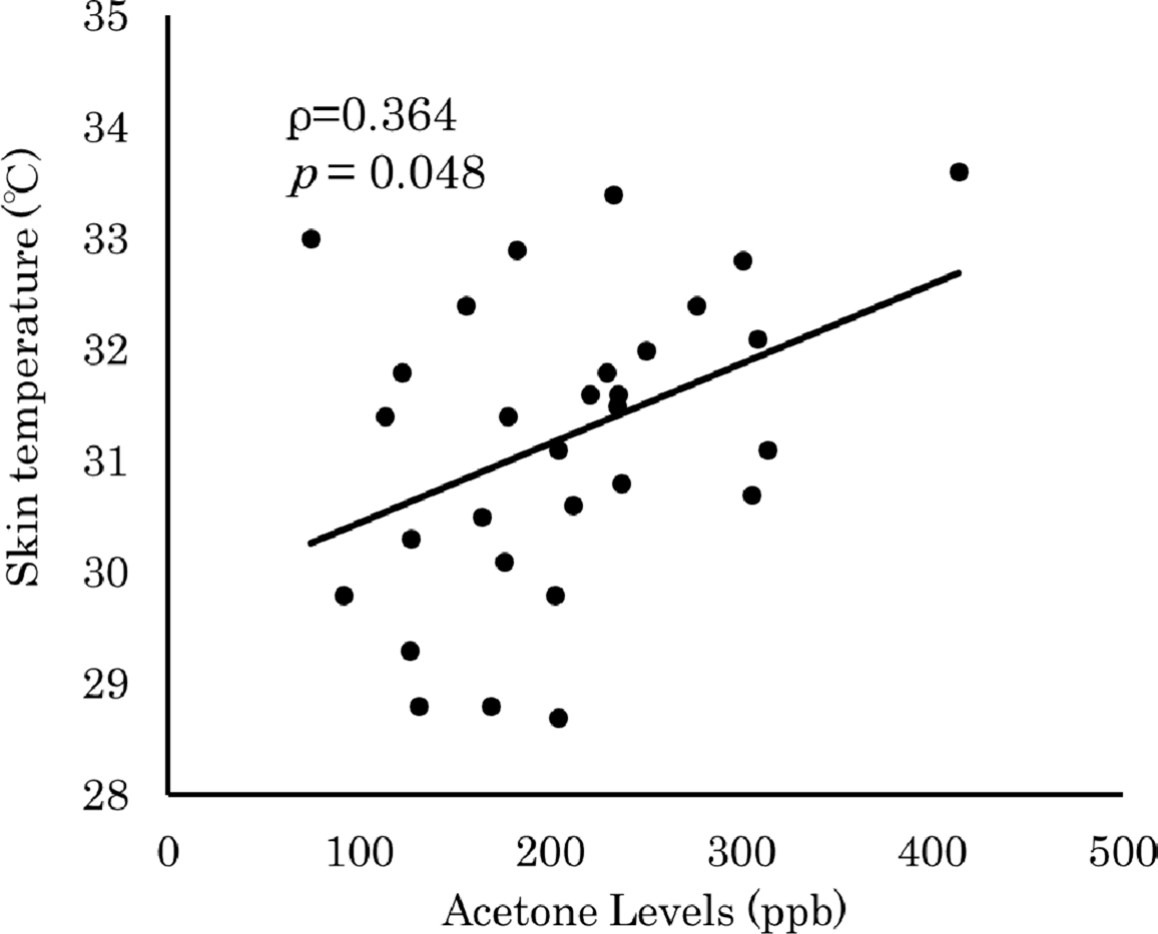
Correlation between skin gas acetone levels and skin temperature at the non-heated site. Measurements obtained at baseline (before heating) and 30 min after local heating were analyzed (*n* = 15). The correlation coefficients and corresponding *p*-value are shown.

At the non-heated skin site, acetone levels showed a significant positive correlation with skin temperature (ρ = 0.364, *p* = 0.048; Fig. 4). No significant correlations were observed with skin blood flow or blood β-hydroxybutyrate concentration. The regression model for the non-heated site was also significant (F (3,26) = 4.208, *p* < 0.015, R² = 0.327). Skin temperature was a significant predictor of acetone levels (β = 0.503, *p* = 0.05), whereas skin blood flow and blood β-hydroxybutyrate concentration were not.

### Relationships between changes in acetone levels and physiological variables

Changes in acetone levels (Δ values, defined as the difference between concentrations after 30 min of heating and at the baseline) and physiological variables before and after heating are summarized in Table 2. At the heated skin site, the changes in acetone levels did not correlate significantly with changes in skin temperature, blood flow, or blood β-hydroxybutyrate concentration. Similarly, at the non-heated skin site, no significant correlations were observed between changes in acetone levels and physiological variables.

**Table 2:** Changes (Δ values) in each parameter at the heated and non-heated sites.

	Heated skin site	Non-Heated skin site
	Mean ± SD	Mean ± SD
Δ Acetone Levels (ppb)	188.8 ± 49.7	80.4 ± 32.1
Δ Skin temperature (℃)	2.4 ± 1.2	1.1 ± 1.2
Δ Skin blood flow (mL/min/100 g)	1.6 ± 1.6	0.6 ± 0.6
Δ Blood β-hydroxybutyrate (mmol/L)	0.0 ± 0.1	0.0 ± 0.1

Values are presented as mean ± standard deviation (SD) (*n* = 15).

## Discussion

In this study, we investigated the effects of local skin heating on acetone levels in skin gas. The results demonstrated that the acetone concentration at the heated skin site increased significantly from 162.9 ± 40.5 ppb at the baseline to 358.0 ± 64.1 ppb after 30 min of heating. Moreover, the acetone levels in the skin gas at the heated site exceeded those in the breath after 10 min of heating. In contrast, no significant changes were observed in the blood β-hydroxybutyrate concentration, an indicator of systemic ketone body metabolism.

Although acetone is primarily eliminated via exhalation^23,24^, our finding that the skin gas acetone levels exceeded the breath levels under local heating does not imply that the skin is a major excretory route.

This observation does not indicate that the total amount of acetone excreted via the skin exceeds that released via breath. Instead, the concentration and total emissions (flux) represent fundamentally different concepts. Although the concentration reflects the local accumulation of acetone near the sampling site, the total emissions depend on both the concentration and volumetric flow rate of the carrier medium. In this study, the total acetone flux from either the breath or skin was not quantified. Therefore, it cannot be concluded that acetone emissions from the skin exceed those from breath under local heating conditions.

Instead, these findings suggest that skin gas acetone concentration can transiently exceed breath levels under specific local thermal and circulatory conditions.

Multiple regression analysis revealed that an increase in the skin gas acetone level at the heated site was strongly associated with an increase in skin temperature. Elevated skin temperatures have been reported to enhance solute diffusion and increase skin permeability by altering the properties of the stratum corneum^25,26^, thereby facilitating the transfer of volatile compounds to the skin surface. In addition, local heating induces capillary vasodilation and increases skin blood flow, thereby enhancing the supply of blood-borne volatile compounds to the skin^27^. In this study, the skin blood flow at the heated site increased during heating, suggesting that changes in skin permeability and increased blood flow, as passive mechanisms, contributed to the observed elevation in skin gas acetone levels. Furthermore, local heating stimulates sweat gland activity and increases the release of VOCs from sweat^28–30^. These factors may act in combination to promote acetone emissions from heated skin. Although alterations in lipid metabolism or localized metabolic activity within skin cells cannot be completely excluded, our data do not provide direct evidence to support such mechanisms. Hence, this interpretation remains speculative.

An increase in skin gas acetone levels was observed at the non-heated skin site and was significantly associated with skin temperature. Local heating is known to not only increase blood flow at heated sites^21^, but also induce systemic circulatory responses, including stepwise increases in limb blood flow and cardiac output, even when heating is locally applied^22^. Thermoregulation is centrally integrated with the hypothalamic reflex mechanisms that adjust blood flow across the body in response to thermal input from the skin^31,32^. Therefore, it is plausible that the increase in skin temperature induced by local heating influenced acetone emissions at the non-heated site via mechanisms similar to those operating at the heated site.

Although an increase in skin temperature at the non-heated site was observed (Table 1), systemic circulatory responses were not directly measured in this study. The above interpretation is based primarily on evidence from the literature, rather than direct measurements. Therefore, although the observed changes at the non-heated site were consistent with thermoregulatory mechanisms, this explanation should be interpreted with caution.

Simple correlation analysis did not reveal any significant association between blood β-hydroxybutyrate concentration and skin acetone levels. Although the blood β-hydroxybutyrate concentration emerged as a significant predictor in the multiple regression model at the non-heated site, corresponding changes were not observed in acetone levels at the heated site or in breath, and blood β-hydroxybutyrate concentration itself did not change significantly. These findings suggest that the observed statistical significance at the non-heated site may be attributable to a suppression effect, rather than a reflection of true systemic metabolic changes.

Skin gas exhibits delayed temporal behavior relative to breath^33^, potentially reflecting skin-specific elimination processes involving diffusion distance, tissue partitioning, and blood flow dependence. Therefore, the increase in skin gas acetone levels observed in this study can be explained by the enhanced re-release of acetone from the skin surface, driven by passive mechanisms, including increased blood flow, facilitated mass transfer, and altered evaporation dynamics of sweat- and sebum-derived components. Importantly, our findings do not provide evidence of an active physiological mechanism by which the skin excretes acetone.

This study had several limitations. First, the relatively small sample size may have introduced variability, limiting the robustness and generalizability of the findings. Although consistent trends were observed, the possibility that some of the observed changes were influenced by random variations cannot be excluded. Further studies with larger sample sizes are required to confirm the reproducibility of these findings. Second, only male participants were included to minimize potential confounding effects related to hormonal fluctuations^34^ and sweating^35^ in thermoregulation and metabolism. Although this approach enhanced the internal validity of this initial mechanistic investigation, the generalizability of our findings to females remains uncertain. Future studies including both sexes are warranted to elucidate potential sex-specific effects. Additionally, active excretion mechanisms and acetone production by skin cells were not directly examined, and the diffusion pathways and storage dynamics within skin tissues remain unclear. Furthermore, whether the heating-induced changes observed in this study are specific to acetone or applicable to other skin gas components requires further investigation. Future studies should examine the effects of local heating on additional skin gas components, and quantitative analyses of intradermal diffusion and storage dynamics are required to further elucidate the physiological basis of skin gas emissions. Despite these limitations, our findings demonstrate that skin gas acetone reflects dynamic, condition-dependent physiological changes and highlight its potential utility for noninvasive metabolic monitoring.

## Conclusions

In this study, we examined the effects of local skin heating on acetone levels in skin gas. The results demonstrated that skin gas acetone responds to local increases in skin temperature and blood flow, and that its levels can exceed breath acetone levels during short-term heating. These findings suggest that acetone in skin gas reflects dynamic, local, physical, and circulatory changes, independent of systemic metabolic alterations. Therefore, this study contributes to a deeper fundamental understanding of skin gas analysis and supports its potential application in noninvasive biomonitoring.

## Methods

### Participants

Twenty-one healthy adult males participated in this study. All participants were free from cardiovascular, metabolic, or dermatological diseases and were not taking any medication at the time of the experiment. The mean age of the participants was 22.4 ± 2.2 years and the mean body mass index (BMI) was 23.9 ± 3.5 kg/m² (mean ± standard deviation).

The experiments were conducted between January and October 2025. The study protocol was approved by the Ethics Committee of Chubu University (approval number: 20240055-2). All procedures were performed in accordance with the Declaration of Helsinki and relevant institutional guidelines and regulations. Written informed consent was obtained from all participants prior to their participation.

### Experimental protocols

#### Pre-experimental controls

The participants were instructed to refrain from alcohol consumption, smoking, and vigorous physical activity from the morning before the experiment until its initiation. To minimize metabolic variability, all participants consumed identical meals at standardized times on the experimental day. The participants consumed steamed white rice (300 g) at 08:00 and a beef bowl meal at 12:00. The nutritional composition of the meals is summarized in Table3. All experiments were initiated at 15:00, 3 h after lunch.

**Table 3:** Nutritional composition of the consumed meals.

	Energy (kcal)	Protein (g)	Fat (g)	Carbohydrate (g)	Sodium (g)
White rice(300 g)	441.0	6.3	0.0	101.7	0.0
Beef bowl	823.0	24.8	29.0	119.5	3.1

Experiments were conducted in a temperature-controlled room maintained at 25 °C. Throughout the experiment, participants remained seated and at rest.

#### Local heating protocol

Local skin heating was applied to the right hand and forearm using an electric heating pad (YMM-W45BTH; Yamazen Corporation, Japan), which was wrapped around the right hand from the fingertips to the distal forearm and secured to maintain close contact with the skin. The local heating temperature was set to 41 °C based on preliminary experiments that confirmed enhanced skin gas release while ensuring participant safety. The temperature was maintained and local heating was continued for 30 min. Heating was initiated immediately after baseline skin gas sampling.

### Skin gas and breath sampling

Skin gas samples were collected using glove-based sampling. A polyvinyl fluoride (Tedlar^®^) bag was custom-shaped to tightly enclose the hand and wrist, allowing for the airtight accumulation of skin-emitted gases. Prior to sampling, participants thoroughly washed their hands with running water to remove any surface contaminants. After drying, the hand was inserted into the sampling bag, and the air inside the bag was evacuated to minimize contamination from ambient air.

After evacuation, the participants remained seated without local heating for 10 min to allow the accumulation of skin-emitted gases; this sample was designated as the baseline. Immediately thereafter, local heating was initiated as described above, and skin gas samples were collected at 10, 20, and 30 min after heating onset. Skin gas was sampled at both the heated (right hand) and non-heated sites, enabling a direct comparison between locally heated and non-heated conditions.

Breath samples were collected concurrently with the skin gas samples. Participants remained seated at rest and breathed normally. After several tidal breaths, end-tidal breaths were collected using an aluminum sampling bag (Collection Bag, Laboratory for Expiration Biochemistry, Nourishment Metabolism Co., Ltd., Nara, Japan) by discarding the initial portion of exhaled air and collecting approximately 400 mL of the terminal phase of expiration.

All procedures were conducted under biosafety level 1 conditions in accordance with the institutional safety protocols.

Throughout this study, all personnel involved in sample handling and analysis wore disposable gloves, masks, and laboratory coats to minimize exposure to biological materials. Each participant used disposable mouthpieces and sampling bags to prevent cross-contamination. Breath samples were handled and analyzed using a closed system to further reduce the risk of exposure.

### Physiological measurements

Physiological measurements were performed in a subset of 15 participants. Skin temperature, skin blood flow, and blood β-hydroxybutyrate concentrations were measured immediately before baseline skin gas and breath sampling and again after completion of the 30-min local heating protocol. These assessments were added after the commencement of the study and were, therefore, not available to all participants. Participants were not selected based on their acetone responses or other outcome measures.

The skin temperature was measured at the surface using a digital thermometer (HA-100E, Anritsu Corporation, Japan). Skin blood flow was assessed using a laser Doppler flowmetry system (ALF21; Admedec Corporation, Japan). Blood β-hydroxybutyrate concentrations were measured using a portable enzymatic analyzer (FreeStyle Precision Neo, Abbott Diabetes Care Inc., Alameda, California, USA) according to the manufacturer’s instructions.

### Gas analysis

Acetone levels in skin gas and breath samples were analyzed using sensor gas chromatography (SGEA-P3-A, Nissha FIS Inc., Japan). Quantification was performed using calibration curves generated from certified standard gases.

A mixed standard gas containing acetone at a certified concentration of 5.21 ppm (CBN1A11, Sumitomo Seika Chemicals Co., Ltd., Japan) was diluted with purified air (NHB 88 − 62, Taiyo Nippon Sanso Corporation, Japan) to prepare calibration standards at five concentrations (100, 200, 500, 1000, and 4000 ppb). Calibration standards were analyzed under identical conditions and calibration curves were constructed to correct the measured values.

To account for potential contributions from ambient acetone, background air samples were collected on the same day and analyzed using the same system. The ambient acetone concentrations were subsequently subtracted from the measured acetone levels in skin gas and breath samples.

### Statistical analysis

Acetone levels were analyzed separately in breath and skin gas samples from heated and non-heated sites. To evaluate time-dependent changes and differences among sampling sites, a two-way repeated-measures ANOVA was conducted with sampling site (breath, heated skin, and non-heated skin) and time (baseline, 10, 20, and 30 min) as within-subject factors. When the assumption of sphericity was violated, Greenhouse–Geisser corrections were applied.

Linear mixed-effects models were used to further compare the time-course changes in skin gas acetone levels between heated and non-heated sites. In these models, acetone levels were treated as the dependent variable, and sampling sites (heated vs. non-heated) and time (baseline, 10, 20, and 30 min; treated as categorical variables) were included as fixed effects, along with their interaction. A random intercept for each participant was included to account for interindividual variability. Breath acetone levels were included as time-varying covariates to control systemic acetone dynamics. The model parameters were estimated using the restricted maximum likelihood method, and the degrees of freedom were calculated using the Satterthwaite approximation. Type III tests were used to evaluate fixed effects. Post hoc comparisons were performed using Bonferroni correction based on the estimated marginal means when significant main effects or interactions were observed.

Changes in physiological variables before and after local heating were assessed using the Wilcoxon signed-rank test. The differences between heated and non-heated sites at the corresponding time points were evaluated using the Wilcoxon signed-rank test. Associations between the skin gas acetone concentrations and physiological variables (skin temperature, skin blood flow, and blood β-hydroxybutyrate concentration) were examined using Spearman’s rank correlation analysis.

Multiple linear regression analysis was conducted with skin gas acetone levels as the dependent variable and skin temperature, skin blood flow, and blood β-hydroxybutyrate concentration as independent variables. Multicollinearity was assessed using the VIF, with values below five considered acceptable.

All statistical tests were two-tailed, and *p*-values < 0.05 were considered statistically significant. Statistical analyses were performed using the IBM SPSS Statistics version 31 (IBM Corp., Armonk, NY, USA).

## Data Availability

The datasets used and/or analyzed in the current study are available from the corresponding author upon reasonable request.
